# Interspecific Hybridization Increased in Congeneric Flatfishes after the *Prestige* Oil Spill

**DOI:** 10.1371/journal.pone.0034485

**Published:** 2012-04-12

**Authors:** Victor Crego-Prieto, Jose L. Martinez, Agustin Roca, Eva Garcia-Vazquez

**Affiliations:** Department of Functional Biology, University of Oviedo, Oviedo, Spain; Institute of Marine Research, Norway

## Abstract

Marine species with relatively low migratory capacity are threatened by habitat alterations derived from human activities. In November 2002 the tanker *Prestige* sank off the Spanish northwest coast releasing 70,000 tons of fuel and damaging biota in the area. Despite efforts to clean the damaged areas, fuel remnants have affected marine species over the last nine years. This study is focused on two flatfish, *Lepidorhombus boscii* (four-spotted megrim) and *L. whiffiagonis* (megrim), whose spawning areas are located at the edge of the continental platform. We have analyzed megrim samples from North Spanish and French waters obtained before and after the oil spill. Genotypes at the nuclear marker 5S rDNA indicate a significant increase in interspecific hybridization after the *Prestige* accident, likely due to forced spawning overlap. The mitochondrial D-Loop region was employed for determining the direction of hybrid crosses, which were most frequently *L. boscii* female x *L. whiffiagonis* male. Reduced ability of *L. boscii* females to select conspecific mates would explain such asymmetric hybridization. To our knowledge this is the first time that increased hybridization between fish species can be associated to an oil spill. These results illustrate the potential long-term effect of petrol wastes on wild fish species.

## Introduction

Many human activities endanger survival of marine species. For example, wild populations are threatened by high ship traffic [1], overfishing [2] and many others, such as oil spill accidents [3]–[5], whose long term consequences have not yet been evaluated. Oil spills cause severe damage to marine wildlife due to polycyclic aromatic hydrocarbons (PAHs) released from fuel remnants causing oxidative stress, tissue alterations and cell death among other injuries by increasing oxygen-derived free radicals [6], ([7] and references therein).

**Figure 1:**
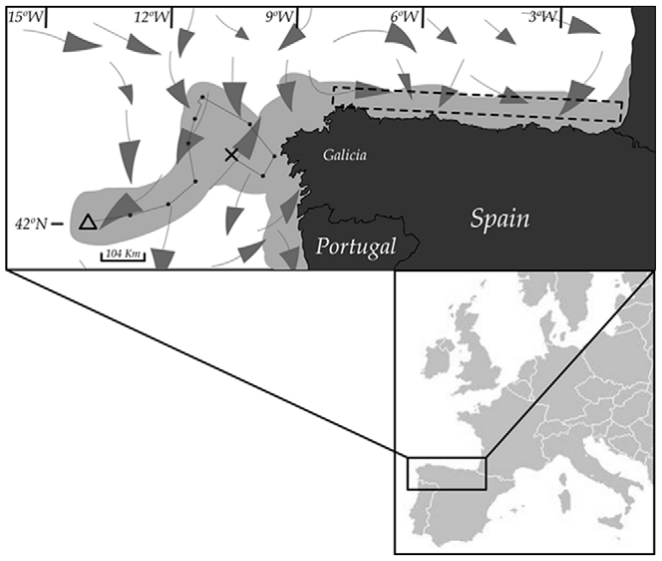
Map of the area affected by the *Prestige* oil spill and sampling area. Map of Northwest Iberia (Cantabric Sea) showing the location where the *Prestige* sunk (triangle off Galician coast) and its last trajectory (black line with dots) from the location of the accident and mayday alarm (cross). Arrows represent marine currents in the area and season of the accident (taken from http://oceancurrents.rsmas.miami.edu/). Their sizes are representative of their strength. The area affected by the oil spill is marked in light grey. The sampling area is marked as a dotted rectangle.

The *Prestige* oil spill occurred off the Galician coast (Northwest of Spain; Fig. 1) on 13^th^ of November 2002, and was classified as one of the worst ecological catastrophes of the century in Europe [8]. The *Prestige* tanker carried about 77,000 tons of fuel type M-100 (one of the most toxic petroleum derivatives), of which 20,000 tons were dumped directly into the sea at the time of the accident affecting an area of 30,000 km^2^ [9]. From then until the summer of 2003, 40,000 more tons were spilled [8] affecting marine communities of the Cantabric Sea and the Bay of Biscay along 2,600 coastal km [10], reaching the oyster farms in the Bay of Arcachon (France). Oil pollution caused a great damage to both marine biodiversity and the local economy [11]. Eight years after the disaster there were still at least 202 beaches contaminated by fuel in Galicia, and many others in Asturias, Cantabria and the Basque Country (www.wwf.panda.org).

Many marine vertebrates were affected by the oil spill including birds, mammals and fish [12], [13] and a review by [14]. Due to the high density of the fuel, it accumulated on the sandy bottom of the continental shelf. Species that swim and live on the bottom where the fuel accumulated were consequently most affected by the oil spill [8], [10], [15]. Previous studies carried out in the Mediterranean Sea demonstrated that the megrim *L. boscii* is a very sensitive species to PAHs exposure [16], and it was also especially affected by *Prestige* PAHs, which altered its DNA integrity and increased levels of stress and genotoxicity biomarkers [10].

*L. boscii* and its congeneric sympatric species *L. whiffiagonis* (Scophthalmidae, Pleuronectiformes) are distributed in the Atlantic Ocean from Iceland to Cape Bojador (26°N), and in the Mediterranean Sea. Both species spawn on the continental shelf from March to June. Little is known about the biology of megrims populations. Sanchez *et al.* [17] suggested low migratory capacity of both megrim species with aggregation and disaggregation movements. Surveys have shown that larvae do not move much from their spawning sites during the first year of life [18]. Spatial genetic differences have been described within their Atlantic area of distribution, megrims inhabiting the Bay of Biscay, Cantabric Sea, Galician and Portuguese coasts belonging to the same population cluster in the two species [19]. Studies on juveniles have been focused on development and growth patterns [17], [20], [21], but megrims' spawning areas have not been studied in depth and the reproductive barriers between the two species are unknown. The age at first maturity is 1.5 years for *L. boscii* [22] and two years could be reasonably considered their generation time; therefore approximately four generations (nine years) were affected since 2002 when the *Prestige* sank. The oil spill was quickly displaced during the 2002–2003 winter and in to a lesser extent until spring and summer 2003 [8] by both surface and deepwater currents, and fuel was deposited on the seabed, most likely reducing suitable megrim spawning areas and thus increasing reproductive interactions between the two species. Furthermore, the seabed was not cleaned up and the oil deposited likely has continued to affect megrim spawning since the spill until the present day. Interspecific hybridization can follow habitat alterations [23]. Our hypothesis is that the habitat alteration due to the *Prestige* accident has forced the two megrim species towards a closer interaction due to the reduction of “clean” spawning areas, especially *L. boscii* because of its higher sensitivity to fuel toxicity [10] and maybe altering their mating behaviour. As a consequence of these factors, together or separately, we would expect increased interspecific hybridization between the two megrim species.

The aim of this study was to test whether interspecific hybridization between the two megrim species increased after the *Prestige* oil spill in the Cantabric Sea, which contained the coastal regions most affected by the catastrophe [9], [10]. The nuclear 5S rDNA locus, frequently used as species-specific marker in fish [24], and RFLPs (Restriction Fragment Length Polymorphisms) at the mitochondrial D-loop sequence for determining the maternal species of hybrids were employed as molecular markers for identifying interspecific hybrids.

## Results

**Figure 2:**
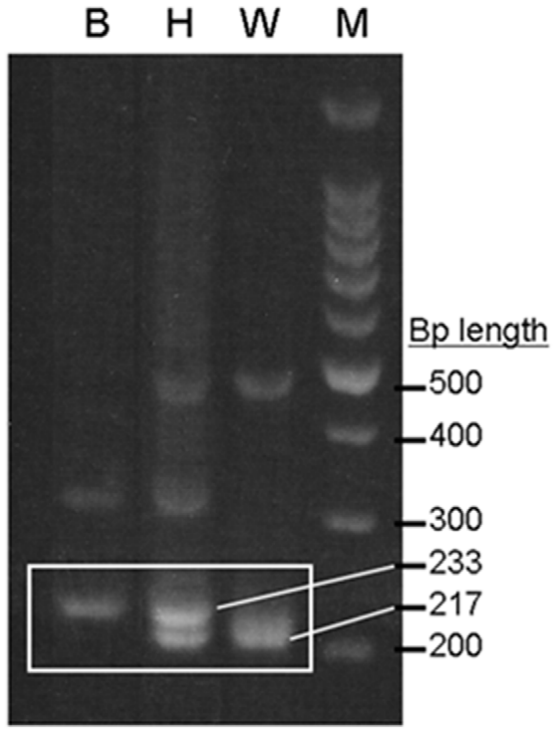
Agarose gel with amplification fragments of the 5S rDNA for pure and hybrid individuals. Agarose gel (2.5%) stained with ethidium bromide containing PCR amplification products of the 5S rDNA of *Lepidorhombus boscii* (B), the interspecific hybrid (H) and *L. whiffiagonis* (W). M: 100 bp ladder as DNA fragment size marker.

Amplification of the 5S rDNA locus yielded different fragments for each species. *Lepidorhombus boscii* exhibits one main fragment 233 bp long and a secondary fragment of 330 bp (much weaker in the gels). For *L. whiffiagonis* we obtained two fragments of 217 (main) and 472 (secondary, also weaker) bp long, as described in [25] for Atlantic megrim. Before the *Prestige* accident, only one individual (0.75%) from the sampled area exhibited a pattern of amplification fragments which corresponded to an interspecific hybrid, containing the two main fragments of each species: 217 and 233 bp long (Fig. 2). The weaker species-specific secondary fragments also appeared but are less clear in agarose gels and were not considered in this study. The hybrid specimen had been classified *de visu* as *L. whiffiagonis*. After the accident the situation changed drastically (Table 1). A total of 38 individuals (25.67%) exhibited hybrid genotypes. Three of them exhibited a typical *L. whiffiagonis* phenotype and 35 were *L. boscii*-like. A Chi-Square analysis confirmed that the proportion of interspecific hybrids increased significantly after the *Prestige* accident (χ^2^ = 36.54, 1 degree of freedom, *P*<<0.001). The species composition of the samples was also different, with more *L. whiffiagonis* in the after-Prestige sample and more *L. boscii* in the before-Prestige one.

**Table 1:** Species identification of megrim samples obtained before and after the *Prestige* accident.

	*L. whiffiagonis*	Hyb Lw	Hyb Lb	*L. boscii*	N
Before *Prestige*	38 (28.4%)	1 (0.7%)	0 (0%)	95 (70.9%)	134
After *Prestige*	98 (66.2%)	3 (2.0%)	35 (23.6%)	12 (8.1%)	148

Results are presented as the number (percent) of individuals. A first classification of individuals in interspecific hybrid or in each of the pure *Lepidorhombus* species was based on the 5S rDNA marker. Afterwards, hybrid individuals were classified as belonging to one of the two main species based on external phenotype and mitochondrial D-loop sequences (maternal species) as Hyb Lw (both *L. whiffiagonis* phenotype and mother) or Hyb Lb (both *L. boscii* phenotype and mother). N: total number of samples.

**Figure 3:**
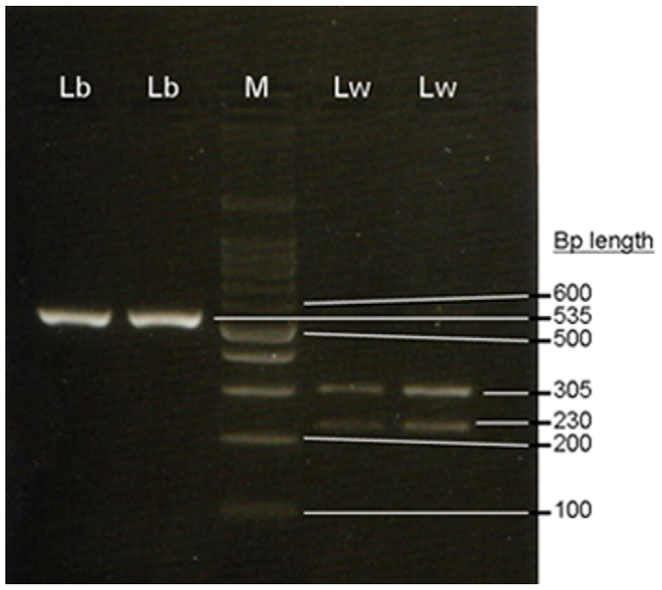
Agarose gel (2.5%) showing the different Dra I patterns for megrims D-loop. Lb: *Lepidorhombus boscii*, M: 100 bp DNA ladder, Lw: *L. whiffiagonis*.

The direction of hybrid crosses was assessed from D-loop RFLP. Amplification of this region with D-loopDF and D-loopDR primers yielded one fragment 535 bp long for both *Lepidorhombus* species. After digestion with *Dra I*, individuals with *L. boscii* mitochondrial D-loop (without restriction targets for this enzyme) yielded the same uncut fragment of 535 bp, as expected. Individuals with *L. whiffiagonis* mitochondrial DNA, with one *Dra I* restriction target, provided two fragments 230 and 305 bp long (Fig. 3) as also expected from the sequences. The 35 hybrids classified *de visu* as *L. boscii* exhibited a *L. boscii* D-loop pattern, and the three hybrids morphologically identified as *L. whiffiagonis* possessed a *L. whiffiagonis* D-loop (Table 1). As mitochondrial DNA is maternally inherited, we can conclude that most of the hybrid crosses that occurred after the *Prestige* accident (92.1% of the hybrids found in this survey) corresponded to *L. boscii* females mating with *L. whiffiagonis* males. All the hybrids were classified *de visu* as belonging to the maternal species, indicating that the external phenotype could be heavily influenced by the mother in interspecific crosses.

## Discussion

As the first long-term investigation of the area, the results found in this study reveal an increase in hybridization between the two megrim species in an area especially affected by the *Prestige* oil spill [9], [10]. The proportion of hybrids changed from less than 1% to more than 25% in only nine years and, according to the D-loop region, most hybrid crosses involved *L. boscii* females and *L. whiffiagonis* males. Increased hybridization between sympatric species following environmental disturbances has been observed in a wide range of plant and animal taxa [26]. For example, the proportion of interspecific hybrids between stickleback species from Enos Lake (Victoria, British Columbia, Canada) increased from 1% [27] to 12% [28] or 24% [29] following an anthropogenic-derived ecological change (introduction of an exotic predator) [30]. A species breakdown has been suggested for sticklebacks [29], [30], [31]; however this not seems to be the case for megrims, in which only part of one area/population of the whole distribution was affected, similar to a unique event of hybridization increase. The appearance of many hybrids in the studied area may not be directly attributed to habitat loss caused by the petrol waste because interspecific matings on affected sea bottoms have not been physically observed. However, it seems to be at least one of the causative factors because loss or alterations of habitats are frequently implicated as contributing factors in hybridization in fishes [32]. In other species such as cichlids, loss of water transparency has caused the rupture of pre-mating barriers based on body coloration, increasing the proportion of hybrids up to 88% [23].

Interspecific megrim hybrids may have accumulated in the studied region after the environmental degradation during the four generations elapsed since the accident. In addition to reduced and deteriorated habitat, and although interspecific mating barriers have not been studied for megrims, stress conditions in the area could have affected the mating behaviour of megrims, which is the second part of our hypothesis. In some amphibians stress affects the quality of male vocalization which is a determinant of female choice [33], [34]. Altered behaviour of females, particularly the rejection of allospecific males, may explain many cases of unidirectional hybridization [35]. For example, rodents of both sexes prefer conspecific over congeneric individuals in normal non-stress conditions, but mate choice may change if the hormonal balance is altered, as happens in stressful conditions [36]. Other studies show that males of high body condition are often preferred by females due to the relationship between body size and male quality or fitness [37], [38]. This could explain the higher proportion of hybrids with a *L. whiffiagonis* father and *L. boscii* mother as *L. whiffiagonis* are larger than *L. boscii*. Although we don't understand the particular underlying mechanism, it is possible that *L. boscii* females were more receptive to accepting males of the other species due to hormonal changes (probably pheromones) in response to the ecological stress caused by the spillage and that those pheromones could act as a pre-mating barrier in non-stressful conditions.

An alternative explanation is that hybrid crosses have not increased but instead the fitness of hybrids has been enhanced. Changes in the fitness of hybrids before and after environmental perturbations have been reported in the scientific literature for both plants and animals [38], [39]. In fish, as in other species, hybrids may constitute a mechanism by which species deal with marginal habitats and environmental deterioration [40] and may even enable colonization of new habitats [41], [42]. Whether hybrid crosses increased directly by forced spawning overlap of the two species when changes in hormones relaxed mating choice, and/or hybrid fitness has increased due to environmental deterioration, the final result was an increase in interspecific hybridization in the area affected by the petroleum.

The difference between pre- and post-*Prestige* samples in the proportion of pure individuals of each species could be explained by differential sensitivity of the two species. Martínez-Gómez *et al*. [10] showed that *L. boscii* was strongly sensitive to the *Prestige* toxic wastes, and the decrease of this species in post-*Prestige* megrim samples is consistent with such high sensitivity, indicating a possible decline in its population size. In addition, the hybridization that occurred after the oil spill was asymmetrical, with a higher proportion of hybrids resulting from *L. boscii* females. The rare species frequently provides the female in hybrid crosses [36], [43], consistent with what we observed in our study with *L. boscii*.

In conclusion, a high increase in interspecific megrim hybrids in the northern Spanish area affected by the *Prestige* oil spill may suggest that the accident could have increased the interspecific hybridization rate between megrims, likely due to a combination of altered mating behaviour and reduction of suitable spawning habitat. These hypotheses should be verified with future work and, if proven correct, the consequences of such interspecific introgression should be examined in further surveys. Although the trace of alien introduced genomes will likely remain for generations, measures for helping the most affected species *L. boscii* including a reduction in fishing mortality by increasing the allowable megrim size at catch, should be considered for future conservation of these valuable flatfish.

**Figure 4:**
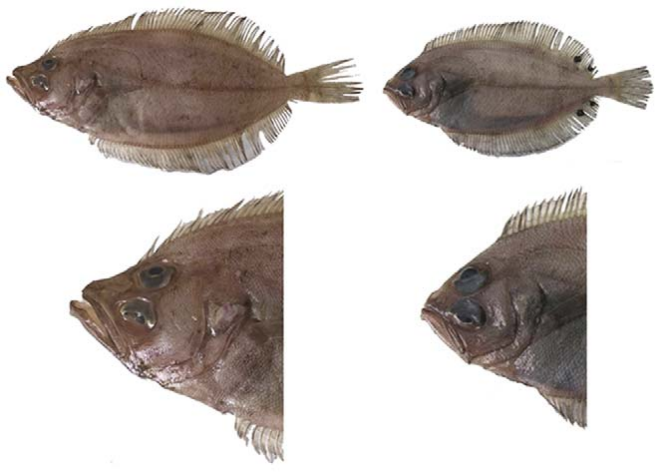
Pictures of megrim species. Pictues of body (up) and head (down) of *Lepidorhombus whiffiagonis* (left) and *L. boscii* (right).

## Materials and Methods

### Samples analyzed

One year before the *Prestige* accident (August 2001) muscle fragments from adults of the two *Lepidorhombus* species were collected during research cruises and identified *de visu* by technical staff of the Spanish research institutions AZTI (a technological centre specialised in marine and food research) and IEO (Spanish Institute of Oceanography) from the Cantabric Sea and Bay of Biscay (corresponding with the ICES area VIIIc). Species classification was made according to head differences between species and the four characteristic spots typical of *L. boscii*'s fins (see Fig. 4). *L. whiffiagonis* individuals present a sharp snout, which is also approximately two-times bigger than their eye diameter, and the dorsal fin origins closer to the tip of the snout than to the anterior edge of the eye. Otherwise, *L. boscii* individuals' dorsal fin originates closer to the anterior edge of eye and presents a smaller snout length than *L. whiffiagonis*. In total 39 *L. whiffiagonis* and 95 *L. boscii* were sampled.

In July 2011, nine years after the oil spill, new adult samples (heads or whole individuals) of the two megrim species were collected randomly from fishing vessels operating across Cantabric Sea and Bay of Biscay waters (similar locations as in 2001). In total we sampled 101 *L. whiffiagonis* and 47 *L. boscii*. They were visually classified as explained above. Only two phenotypic morphs were found across all individuals (corresponding with *L. boscii* and *L. whiffiagonis* “typical” individuals) and no morphological differences were found among individuals belonging to the same species.

A piece of gill or muscle tissue (approx. 3 g) was taken from each sample and stored in 100% ethanol for genetic analyses.

### Genetic analyses

Total genomic DNA was extracted following a Chelex based protocol [44] and stored at 4°C. We amplified the 5S rDNA locus employing the primers A (5′-TACGCCCGATCTCGTCCGATC-3′) and B (5′-CAGGCTGGTATGGCCGTAAGC-3′) designed by [24] in 20 µl of total volume containing 4 µl of 5× Promega Green Buffer, 2 µl of 25 mM MgCl_2_, 2 µl of a 2.5 mM dNTPs mixture, 1 µl of each primer at 20 µM, 0.1 µl of GoTaq polymerase at 5 U/µl (Promega), 2 µl of sample DNA and 7.9 µl of bidistilled water. PCR amplification cycles were: 5 min of initial denaturing at 95°C, followed by 35 cycles of denaturing at 95°C for 20 s, annealing at 65°C for 20 s and extension at 72°C for 30 s, plus a final extension at 72°C for 20 min. Amplification products were run in 2.5% w/v agarose gels at 100 V, and stained with 2 µl ethidium bromide (10 mg/ml) to visualize them. Fragment sizes were estimated by comparison with a standard 100 bp DNA marker (Promega).

The maternal species of hybrids was identified by a species-specific RFLP within the mitochondrial D-loop sequence. To develop the method, D-loop sequences of the two megrim species were obtained from GenBank (accession numbers FJ590680-FJ590700 and FJ590730-FJ590750) and aligned with the ClustalW application [45] included in BioEdit. Invariant (monomorphic) regions were visually identified within each megrim species with the BioEdit Sequence Alignment Editor software [46] after sequences alignment. Restriction enzyme targets within the invariant regions of each species were detected with the NEBcutter ver. 2.0 software. The enzyme *Dra I* recognizes the sequence 5′-TTTAAA-3′ and makes a blunt cut 5′-TTT/AAA-3′ [47]. Such sequence is present in *L. whiffiagonis* and absent in *L. boscii* D-loop sequences.

D-loop amplification was carried out employing the primers D-loopDF (5′-GTCGCCACCATTAACTTATGC-3′) and D-loopDR (5′-CCCAAACTCCCAAAGCTAAG-3′) described by [48]. The amplification mixture, of a total volume of 20 µl, contained 4 µl of 5× Promega Green Buffer, 1.2 µl of 25 mM MgCl_2_, 2 µl of a 2.5 mM dNTPs mixture, 1 µl of each primer at 20 µM, 0.12 µl of GoTaq polymerase at 5 U/µl (Promega), 2 µl of sample DNA and 8.68 µl of bidistilled water. Eight µl of PCR amplification were loaded in a 2% agarose gel stained with 2 µl of 10 mg ml^−1^ ethidium bromide to verify that only one band was amplified. Then 10 µl of the PCR product were mixed with 2.5 µl of 10× Buffer M (Roche), 0.1 µl of Dra I enzyme (Roche) at 10 ud/µl and 12.4 µl of bidistilled water making a total volume of 25 µl, and incubated at 37°C for one hour. After incubation, the products were loaded in a 2.5% agarose gel, run at 80 v for 40 min and stained with 2 µl of 10 mg ml^−1^ ethidium bromide. Fragment sizes were estimated with a standard 100 bp DNA marker (Promega).

### Statistical analysis

The proportions of interspecific hybrids before and after the *Prestige* accident were compared employing a Chi-square contingency test (χ^2^) by hand. The null hypothesis (H_0_) was that the proportions are similar at a confidence level of 95%.
